# The Passalidae (Coleoptera, Scarabaeoidea) from Bolivia, with the descriptions of three new species

**DOI:** 10.3897/zookeys.882.35532

**Published:** 2019-10-23

**Authors:** Larry Jiménez-Ferbans, Pedro Reyes-Castillo, Jack C. Schuster

**Affiliations:** 1 Universidad del Magdalena, Facultad de Ciencias Básicas. Carrera 32 No 22–08, Santa Marta, CZ-470004, Colombia Universidad del Magdalena Santa Marta Colombia; 2 Instituto de Ecología, A.C., Red de Biodiversidad y Sistemática. Xalapa, CZ- 91070, Mexico Red de Biodiversidad y Sistemática El Haya Mexico; 3 Universidad del Valle de Guatemala. Guatemala City, Guatemala Universidad del Valle de Guatemala Guatemala Guatemala

**Keywords:** bess beetles, Central South America, diversity, synopsis, pasálidos, Suramérica central, diversidad, sinopsis

## Abstract

Employing data from literature, examination of specimens in collections, and a field trip, a list of the species of Passalidae from Bolivia is elaborated. A total of 38 species is reported, including new records of *Passalus inca* Zang, 1905 and *P. lunaris* (Kaup, 1871), and three new brachypterous species: *Passalus bolivianus***sp. nov.**, *P. canoi***sp. nov.**, and *P. gonzalezae***sp. nov.** Most of the species (27) belongs to the Passalini tribe, especially to the genus *Passalus* Fabricius, 1792 (19 species); the Proculini tribe is represented by eleven species in three genera. The number of species of Bolivia is low and reflects the lack of a systematic exploration of this country; more surveys are needed, especially in ecosystems such as montane forest and tropical rain forest.

## Abstract (es)

Empleando datos de literatura, el examen de especímenes en colecciones y recolectados en campo, elaboramos una lista de las especies de Passalidae de Bolivia. Registramos un total de 38 especies, incluidos los nuevos registros de *Passalus inca* Zang, 1905 y *P. lunaris* (Kaup, 1871), y tres nuevas especies braquípteras: *Passalus bolivianus***sp. nov.**, *P. canoi***sp. nov.** y *P. gonzalezae***sp. nov.** La mayoría de las especies (27) pertenecen a la tribu Passalini, especialmente al género *Passalus* Fabricius, 1792 (19 especies); la tribu Proculini está representada por 11 especies de tres géneros. El número de especies registradas para Bolivia es bajo y refleja la falta de una exploración sistemática de este país. Se necesitan más muestreos, especialmente en ecosistemas como el bosque montano y la selva tropical.

## Introduction

Passalidae is a Pantropical group of Coleoptera. With few exceptions, the species of the family live inside rotting logs, feeding on decomposing wood. In the New World the family is represented by the tribes Passalini and Proculini, and in South America the majority of the species belongs to Passalini.

Zang (1905) described *Veturius spinipes*, constituting the first record of a Passalidae from Bolivia. After that, Gravely (1918) recorded three species and Luederwaldt (1931a) described *Paxillus pleuralis* from La Paz. Subsequently, other authors have cited and described additional species from Bolivia. Here we compile these records into a single annotated checklist that includes bibliographic references and general comments. Three new species from Bolivia are also described.

## Materials and methods

Pedro Reyes-Castillo conducted a field trip to Santa Cruz in February 2010 and the material collected is deposited in the collection of the Instituto de Ecología in Xalapa (**IEXA**, Mexico). We examined the material from Bolivia deposited in this collection and also from the Museu de Zoologia, Universidade de São Paulo (**MZSP**, Brazil), Universidad del Valle de Guatemala (**UVGC**, Guatemala), The Field Museum of Natural History (**FMNH**, USA), the Colección Entomológica Universidad del Magdalena (**CEBUMAG-ENT**, Colombia) and the Colección Entomológica del Instituto de Ciencias Naturales of Universidad Nacional de Colombia (**ICN**, Colombia). The material was identified by us employing original descriptions, keys, and diagnoses provided in Kuwert (1898), Luederwaldt (1931a, b), Hincks (1940), Marshall (2000), Gillogly (2005), Boucher (2006), and Jiménez-Ferbans et al. (2013, 2016), and by comparison to the reliably identified material housed in IEXA and UVGC. In addition to the museum specimens, we reviewed the publications regarding the records of Passalinae from Bolivia.

For every species in the list, we included the entomological collection where the specimens from Bolivia are deposited, the authors that have recorded the species, the material examined (labels cited verbatim and separated by slashes), and comments. The classification adopted and the terminology employed for the head is that proposed by Boucher (2006), for the rest of the body that of Reyes-Castillo (1970).

## Results

A total of 22 species has been recorded from Bolivia in the literature; meanwhile we found 25 species in the reviewed collections, including the specimens of *Passalus inca* from Conchabamba, Yungas del Palmar and *P. lunaris* from Santa Cruz, Chiquitos, new records for Bolivia, and specimens of 3 new species described below.

### Annotated list of the Passalidae from Bolivia

#### Proculini

**1. *Popilius marginatus* (Percheron, 1835)**

*Popilius marginatus* (Percheron, 1835): Gravely (1918: 27), Hincks and Dibb (1935: 18), Doesburg (1942: 330), Gillogly (2005: 84).

**Material examined. Bolivia**: Guanay. X-1992. sp49. M. Kon, leg. 2004 // *Popilius* sp. ca *marginatus* (Percheron) Reyes-Castillo, det. 2006 (1 IEXA). Santa Cruz, Chajare (San Antonio) (1 IEXA). Sierra Santa Ana (1 IEXA). Santa Cruz. 4–6k SSE Buena Vista. F. & F. Hotel. Nov. 1–8 2002. J.E. Wappes (1 IEXA).

**Comments.** described from Brazil, this species is, according to Gillogly (2005), distributed throughout the Amazon Basin. It has been recorded from Argentina, Bolivia, Brazil, Colombia, French Guiana, Peru and Suriname (Hincks and Dibb 1935; Gillogly 2005; Jiménez-Ferbans et al. 2013).

**2. *Popilius tetraphyllus* (Eschscholtz, 1829)**

*Popilius tetraphyllus* (Eschscholtz, 1829): Gillogly (2005: 96).

**Comments.** described from Guiana, this species has a South American distribution that includes Bolivia, Brazil, Colombia, French Guiana, Guyana, Tobago, and Venezuela (Gillogly 2005; Jiménez-Ferbans et al. 2015). Gillogly (2005) recorded a specimen from “Bolivia. Beni: Chalcobo Indian Village (on Rio Benicito) (FMNH)”.

**3. *Verres furcilabris* (Eschschltz, 1829)**

*Verres furcilabris* (Eschschltz, 1829): Hincks and Dibb (1935: 29), Doesburg (1942: 330), Marshall (2000: 45), Boucher (2006: 352).

**Material examined. Bolivia**: Departamento de Cochabamba, Prov. Chapare, Sn. Antonio. IV-1953. Alt. 400 m. A. Martínez Col. // Selva tipo Amazónico (1 IEXA). Guanay. Sp48. X-1992. M. Kon leg. 2004. // *Verres furcilabris* (Eschscholtz) P. Reyes Castillo, det. 2005 (1 IEXA). Dpto. Santa Cruz, Prov. Florida, Samaipata, Paredones. 18 Nov 06. 18°8.437'S, 63°48.131'W. Altitud 1730 m. Cultivo abandonado (chaco Viejo). P. Reyes Castillo, col. // *Verres furcilabris* (Eschscholtz) P. Reyes Castillo, det. 2008 (1 IEXA). Dpto. Santa Cruz, Prov. Sara, Santa Rosa. XI-69. A. Martínez col. // *Verres furcilabris* (Eschscholtz) P. Reyes Castillo, det. 78 (1 IEXA). Sara, Santa Rosa, XI 1969, A. Martínez (1 IEXA). San Jose de Uchupiamonas. Alto Limon. 900 m. 19/30.VIII.96. col. A. Lopera B.H.T E.H. // *Verres furcilabris* Esch. Det. Amat 2001 (1 ICN-ENT).

**Comments.** described from Guiana, this species is distributed in Bolivia, Brazil, Colombia, Ecuador, French Guiana, Guyana, Peru, Suriname, Trinidad and Tobago, and Venezuela (Hincks and Dibb 1935; Marshall 2000; Ratcliffe et al. 2015).

**4. Veturius (Veturius) boliviae Gravely, 1918**

Veturius (Veturius) boliviae Gravely, 1918: Gravely (1918: 38), Hincks and Dibb (1935: 24), Doesburg (1942: 330), Boucher (2006: 468).

**Material examined. Bolivia**: // ex coll H. Boileau. 1925. // *Veturius boliviae* Gravely (1918). S. Boucher det. 1988 (1 IEXA). Dpto. Cochabamba, Prov. Carrasco, Yungas. II-1971, Alt. 3200 m. A. Martínez col. // *Veturius boliviae* Gravely (1918). S. Boucher det. 04 // 266 (1 IEXA). Same data // Bosque Húmedo de Montaña. Bosq. de *Podocarpus* // *Veturius boliviae* Gravely (1918). S. Boucher det. 1988 (1 IEXA). Cochabamba, Carrasco, Khara Huasi 1880–1 900 m, E.N. Smith XII.1991 (3 UVGC). Santa Cruz, Florida, Samaipata, Abra de los Toros. 18 Nov. 2006. 18°7.113'S, 63°48.054'W. Altitud 2030 m. Bosque de lauráceas y helechos arborescentes. P. Reyes Castillo, col. // *Veturius boliviae* Gravel. P. Reyes-Castillo, det. 2008 (6 IEXA).

**Comments.**Gravely (1918) described this species from five specimens from “Chaco, Bolivia”. Boucher (2006) considered it as endemic to the Andes of Bolivia.

**5. Veturius (Veturius) dreuxi Boucher, 2006**

Veturius (Veturius) dreuxi Boucher, 2006: Boucher (2006: 470).

**Comments.**Boucher (2006) described *V. dreuxi* from Bolivia and Paraguay, citing the material from Bolivia as “Bolivie, La Paz, Nor Yungas, Pucara près Caranavi, 850 m, piège lumineux, P. Bleuzen & G. Lecourt X.1993 (MNHN). Bolivie, La Paz, Nor Yungas, Incahuara près Caranavi, 1500 m, piège lumineux, G. Lecourt XI.1991 (MNHN); Bolivia, Coroico [Nor Yungas] // Ex. Staudinger & Bang Haas (MUHD); Bolivia, Yungas de La Paz (MNHB)”. Until now, this species is only known from the type material.

**6. Veturius (Veturius) guntheri Kuwert, 1898**

Veturius (Veturius) guntheri Kuwert, 1898: Kuwert (1898: 173), Hincks and Dibb (1935: 25), Doesburg (1942: 330), Boucher (2006: 440).

**Comments.**Kuwert (1898) described this species based on specimens from “Mons Sorato in Bolivia”. Recently, Boucher (2006) proposed *V. platyrrhinoides* Kuwert (Bolivia), *V. peruvianus* Arrow (Peru) and V. platyrhinus var. fassli Luederwaldt (Ecuador) as synonyms of *V. guntheri*. Thus, the distribution of the species includes Bolivia, Ecuador and Peru.

**7. Veturius (Veturius) libericornis Kuwert, 1891**

Veturius (Veturius) libericornis Kuwert, 1891: Hincks and Dibb (1935: 25), Boucher (2006: 472).

**Comments.**Kuwert (1891) described *V. libericornis* from the Amazon region, without more precision. This species has been recorded from Bolivia, Brazil, Ecuador and Peru (Hincks and Dibb 1935; Boucher 2006; Ratcliffe et al. 2015). Boucher (2006) cited material from Bolivia as “Bolivie, La Paz, Nor Yungas, Incahuara près Caranavi, ± 850 m, piège lumineux, G. Lecourt XI.1991-XI.1992 (MNHN) ; Bolivie, La Paz, Iturralde, rte Rurrenabaque – Ixiamas, 400 m, piège lumineux, P. Bleuzen & G. Lecourt X.1993 (MNHN); Bolivie, La Paz, Nor Yungas, rte Pucara à Caranavi, 850 m, piège lumineux, P. Bleuzen & G. Lecourt X.1993 (MNHN); Bolivie, La Paz, Nor Yungas, rte Caranavi à Carrasco, 1260 m, piège lumineux, G. Lecourt XII.1995 (MNHN); Bolivia, Coroico / *V. libericornis* Kuw. ?, det Hincks (MUHD 1 ex); Bolivia, Yungas de la Paz (MNHB 2 ex). – Bolivie, Santa Cruz, Buena Vista, P. Steinbach (MNHN)”.

**8. Veturius (Veturius) libericornis Kuwert, 1891**

Veturius (Veturius) libericornis Kuwert, 1891: Boucher (2006: 486).

**Comments.** described from Brazil, Boucher (2006) reports it from Argentina, Bolivia, Brazil, Colombia, Ecuador, French Guyana, Guiana, Paraguay, Peru, Suriname, Trinidad and Tobago and Venezuela. From Bolivia, Boucher (2006) cited material as “La Paz, Teoponte, Rio Kaka, 400 m, Balogh, Mahunka, Zicsi // XII.1966 // *V. boliviae* Gravely, det. Endrödi 1971 (MTMA). – BENI : Bolivia, Valle del Mamoré, 450 m, XI.1948 (MNHN) ; Bolivia, Beni, B. Malkin VII–VIII.1960 // Chacobo Indian Village on Rio Benicito (FMNH); Bolivia, Beni, Rurrenabaque env., S. & P. Pokorny XI.1998 (CSP)”.

**9. Veturius (Veturius) standfussi Kuwert, 1891**

Veturius (Veturius) standfussi Kuwert, 1891: Hincks and Dibb (1935: 25, as synonym of *V. platyrhinus*), Boucher (2006: 427).

**Comments.** originally described from Venezuela, this species is distributed in the Andes of Bolivia, Colombia, Ecuador, Peru and Venezuela (Boucher 2006). Boucher (2006) cited localities from Bolivia as “Bolivie, Riv. Songo, A.H. Fassl (MNHN); Bolivie, Nor Yungas, Incahuara près Caranavi, 1500 m, piège lumineux, G. Lecourt XI.1991 (MNHN); Bolivie, Caranavi, 1500 m, piège lumineux, G. Lecourt XI.1992 (MNHN); Bolivie, près Caranavi, env. 1 000 m, X.2002 (MNHN); Bolivia, Yungas de La Paz [A. Fassl 1912–13] (MNHB 1 ex); Bolivia, Coroico // Ex. Staudinger & Bang Haas (MUHD); Bolivie, La Paz, Pucara près Caranavi, 850 m, piège lumineux, P. Bleuzen & G. Lecourt X.1993 (MNHN). –Bolivie, Cochabamba, > 2 000 m, piège lumineux, G. Lecourt X.1990 (MNHN)”.

**10. Veturius (Veturius) yahua Boucher, 2006**

Veturius (Veturius) yahua Boucher, 2006: Boucher (2006: 442).

**Material examined. Bolivia**: Dpto. Santa Cruz. Prov. Ichilo, Buenavista (Tacú), Alt. 450 m. 6-III-1951. A Martinez, col. // *Veturius* (*V.) yahua*. M. PARATYPE. S. Boucher det. 04 // PARATYPE (2 IEXA).

**Comments.**Boucher (2006) described *V. yahua* from Bolivia, Brazil, Colombia, Ecuador, and Peru.

**11. Veturius (Publius) spinipes (Zang)**

Veturius (Publius) spinipes (Zang): Zang (1905: 231), Hincks and Dibb (1935: 30), Doesburg (1942: 330), Boucher (2006: 524).

**Material examined. Bolivia**: Chapare. II. 959. Martínez // *Publius crassus* Sm. P. Pereira det. 60 // *Publius spinipes* Zang 1905. S. Boucher det. 89 (1 IEXA).

**Comments.** described by Zang (1905) from “Bolivia, Mapiri”, this species has been recorded also from Peru (Boucher 2006; Ratcliffe et al. 2015). Boucher (2006) also cited material from La Paz, Cochabamba, and Santa Cruz.

#### Passalini

**12. *Paxillus leachi* MacLeay**

*Paxillus leachi* MacLeay: Gravely (1918: 49), Luederwaldt (1931b: 69), Hincks and Dibb (1935: 35, 36 as *Paxillus brasiliensis* and *P. leachi*), Doesburg (1942: 331 as *P. brasiliensis*), Jiménez-Ferbans and Reyes-Castillo (2015: 433).

**Material examined. Bolivia**: Dpto. Santa Cruz. Prov. Ichilo, Buenavista, Tacú, 6-III-1951, A. Martínez (5 IEXA).

**Comments.** this species is distributed throughout the American continent, from Mexico to Argentina.

**13. *Paxillus forsteri* Luederwaldt, 1927**

*Paxillus forsteri* Luederwaldt, 1927: Hincks (1934: 270), Hincks and Dibb (1935: 36).

**Comments.** Described from “Caminas (Goyas)” in Brazil (Luederwaldt 1927), this species is also known from Bolivia and Peru (Hincks and Dibb 1935). Hincks (1934) recorded specimens from “Coroico: Bolivia”.

**14. *Paxillus pleuralis* Luederwaldt, 1931**

*Paxillus pleuralis* Luederwaldt, 1931: Luederwaldt (1931a: 64), Hincks and Dibb (1935: 37), Doesburg (1942: 331), Mattos and Mermudes (2013), Jiménez-Ferbans and Reyes-Castillo (2015: 434).

**Material examined. Bolivia**: Los Molinos, 2000m, 17-VIII-1980 // Comparado con holotipo // *Paxillus pleuralis* Luederwaldt Reyes-Castillo, det. 1988 (1 IEXA). Dpto. La Paz, Bez. Süd-Yungas, Lambate hahe Chulumani, 1600 m // Ch. Bock leg. XI- 1916, ded. 12 8. 1921 // *Paxillus pleuralis* Lueder. det. 31 // 06425 // *Paxillus pleuralis* Luederwaldt 1931, holotipo, Reyes-Castillo, det. 1988 (1 MZSP).

**Comments.** This species was described by Luederwaldt (1931a) from Bolivia; Jiménez-Ferbans and Reyes-Castillo (2015) extended its range to Peru.

**15. *Paxillus camerani* (Rosmini, 1902)**

*Paxillus camerani* (Rosmini, 1902): Jiménez-Ferbans and Reyes-Castillo (2015: 432).

**Material examined. Bolivia**: Dpto. Cochabamba. Prov. Chapares, S.F. del Chipisi, 400 m, IV-1953, Martínez (2 IEXA). Same data // ICN-7078 (ICN-ENT).

**Comments.** this species is from the Amazon Basin: Bolivia, Brazil, Colombia, Ecuador, French Guiana, and Peru (Hincks and Dibb 1935; Amat-García et al. 2004; Mattos and Mermudes 2013). Jiménez-Ferbans and Reyes-Castillo (2015) recorded *P. camerani* for the first time from Bolivia, citing the two specimens from Cochabamba studied here.

**16. *Paxillus martinezi* Jiménez-Ferbans & Reyes-Castillo, 2015**

*Paxillus martinezi* Jiménez-Ferbans and Reyes-Castillo, 2015: Jiménez-Ferbans and Reyes-Castillo (2015: 428).

**Material examined. Bolivia**: Dpto. Cochabamba. Prov. Carrasco, Khora Huasi, 1880–1900 m, 30-XII-91-8-I-92, B.N. Smith // *Paxillus pentaphyloides* Lued. Det.: J. Schuster, 1993 // *Paxillus borellii* (Pangella) Det.: J.C.S. 1999 // Paratype (2 UVGC). Dpto. Cochabamba. Yungas del Palmar, 2000m, III-63, A. Martínez // Paratype (1 IEXA). Chapare. Paratipo: 2200 m, 2-3-II-76 // Achat Pena // Pedro 92 No 3 // Paratype (2 UVGC). Dpto. Santa Cruz. Prov. Florida. El Chape, 1990–2250 m, 8-XII- 91, B.N. Smith // Paratype (1 IEXA). Dpto. Santa Cruz. Prov. Florida. Samaipata: Abra de los Toros, 18°7.113'S, 63°48.054'W, 2030m, 18-XI-2006, Bosque de lauráceas y helechos arborescentes, P. Reyes-Castillo // Holotype (1 IEXA). Same data // Paratype (2 IEXA). Same data // *Paxillus pleuralis* Luederwaldt, P. Reyes-Castillo, det. 2008 // Paratype (2 IEXA).

**Comments.** described from Bolivia, this species is only known by the type material.

**17. Passalus (Mitrorhinus) lunaris (Kaup, 1871)**

**Material examined. Bolivia**: Dpto. Santa Cruz, Prov. Chiquitos, Santiago de Chiquitos, río Tucavaca 18°18'45.2"S, 59°33'0.4"W, 16.xi.2008 Alt. 319 m // Bosque seco chiquitano, Bajo corteza W.D. Edmonds, P. Reyes, T. Vidaurre, cols. // Passalus (Mitrorhinus) lunaris (Kaup, 1869) Reyes-Castillo, det. 2010 (4 IEXA). Dpto. Santa Cruz, Prov. Chiquitos, Santiago de Chiquitos-Rio Tucavaca 18°16'9.7"S, 59°31'0.7"W 19.xi.2008. Alt. 360 m // Bosque seco chiquitano. En galería inicial, dentro de tronco W.D. Edmonds, P. Reyes, T. Vidaurre, cols. // Passalus (Mitrorhinus) lunaris (Kaup, 1869) Reyes-Castillo, det. 2010 (2 IEXA). Santa Cruz, Florida, Samaipata, río Paredones 19.xi.2006, 18°8.937'S, 63°48.792'W, Altitud 1390 m P. Reyes Castillo, col. (3 IEXA). Dpto. Santa Cruz, 4-6 SSE Buena Vista FandF Hotel 27–29.x.2000 Wappes and Morris // Passalus (M.) lunaris Kaup Mattos det 2014 (1 IEXA). Dpto. Santa Cruz, Reserva Nat. Potrerillo de Guenda 16–22.x.2006, Wappes, Nearns and Ella (1 specimen, IEXA). Prov. Inchilo [Ichilo] Buenavista I. 1950 A. Martínez leg. // Passalus (M.) lunaris Kaup. Mattos det 2014 (1 IEXA). Sp50 M. Kon, leg. 2004. Guanay, Bolivia xi.1992 // Passalus (Mitrorhinus) lunaris (Kaup) Reyes Castillo, det. 2004 (1 IEXA).

**Comments.** Described from Brazil, Luederwaldt (1931b) recorded it from “Campinas (Goyaz)”. Fonseca and Reyes-Castillo (2004: 17) recorded it from the states of Amazonas, Pará, Goiás and Sao Paulo. Outside of Brazil, it has been recorded from Argentina by Bruch (1942) and Jiménez-Ferbans et al. (2013). This is the first record for Bolivia.

**18. Passalus (Pertinax) catharinae Gravely, 1918**

Passalus (Pertinax) catharinae Gravely, 1918: Hincks and Dibb (1935: 43).

**Comments.** This species was described by Gravely (1918: 55) based on two specimens, one from “Santa Catharina” and the other from “Chaco”, without more precision. Hincks and Dibb (1935: 43) assumed “Chaco” as Chaco, Bolivia. We believe nobody has examined specimens of this species after its description.

**19. Passalus (Pertinax) convexus Dalman, 1817**

Passalus (Pertinax) convexus Dalman, 1817: Boucher (1990: 354).

**Material examined. Bolivia**: Dpto. Santa Cruz, Prov. Ichilo, Buenavista, 6.III.951. Alt. 450 m. A Martinez, col. (3 IEXA). Santa Cruz, Prov. Ichilo, Loc. Yapacani (BEEM). 8.VIII.2006 // Leg. I. Garcia, Ma. Julieta Ledezma et al. (3 IEXA). Depto. Beni, Rurrenabaque erea. I-2006. Alt. 230 m. M Kon, col. (1 IEXA). Chajare. II.1952. Antonio Martínez // Passalus (Pertinax) convexus Dalm., P. Pereita det.96 (1, MZSP).

**Comments.** Species with a broad distribution in South America, Boucher (1990) recorded specimens from Cochabamba and Santa Cruz, Bolivia; it has been recorded also from Argentina, Brazil, Colombia, Ecuador, French Guiana, Guyana, Peru, Trinidad and Tobago, Suriname and Venezuela (Luederwaldt 1931b; Hincks and Dibb 1935; Boucher 1990; Amat-García et al. 2004). Luederwaldt (1931b) erroneously recorded it from Chile.

**20. Passalus (Pertinax) morio Percheron, 1835**

Passalus (Pertinax) morio Percheron, 1835: Hincks and Dibb (1935: 45), Doesburg (1942: 331), Luederwaldt (1931b).

**Comments.** Described from Brazil, this species is broadly distributed in South America: Bolivia, Brazil, Colombia, Guiana, Paraguay, Suriname and Argentina (Hincks and Dibb 1935; Doesburg 1942). Luederwaldt (1931b) cited a specimen as “Museu Berlim-Dahlem: Yungas de la Paz (Bolivia) 1000 m”.

**21. Passalus (Pertinax) nodifrons Dibb, 1948**

Passalus (Pertinax) nodifrons Dibb, 1948: Dibb (1948: 284); Hincks and Dibb (1958: 16).

**Comments.**Dibb (1948) described this species citing the following information: “Bolivia: La Paz, received, xii.1928, H. Clemens. Type and paratype (same data) in United States National Museum Collection, Washington”. Until now, nobody has cited more specimens of it.

**22. Passalus (Pertinax) rhodocanthopoides (Kuwert, 1891)**

Passalus (Pertinax) rhodocanthopoides (Kuwert, 1891): Hincks (1949: 58, as *Paxillus tumupasae*), Hincks and Dibb (1958: 17).

**Material examined. Bolivia**: San José de Uchupiamonas, Pie Eslabón, 1200 msnm, 20.vii.1996. Col: A. Lopera B.H.T. // ICN-7085 // Passalus (Pertinax) rhodocanthopoides (Kuwert) det.: Reyes-Castillo 1998 (6 ICN-ENT).

**Comments.** In the catalogue of Hincks and Dibb (1935), this species is recorded from Brazil, Peru, and Suriname. Hincks (1949) described *Paxillus tumupasae* based on specimens from Bolivia; however, Hincks and Dibb (1958) synonymized it with *Passalus rhodocanthopoides*.

**23. Passalus (Passalus) abortivus Percheron, 1835**

Passalus (Passalus) abortivus Percheron, 1835: Hincks and Dibb (1935: 50).

**Material examined. Bolivia**: Buenavista, Ichilo, Santa Cruz. I.49[1949]. A. Mtz [Martínez] Col. // Passalus (Passalus) abortivus Perch. Det.: Jiménez-Ferbans 2016 (1 IEXA).

**Comments.** Species with a Guyano-amazonian distribution, present in Bolivia, Brazil, Colombia, French Guiana, Guyana, Peru, Suriname, Trinidad and Tobago, and Venezuela (Luederwaldt 1931b; Hincks and Dibb 1935; 1958, Reyes-Castillo 1973; Amat-García et al. 2004).

**24. Passalus (Passalus) armatus Perty, 1890**

Passalus (Passalus) armatus Perty, 1890: Hincks (1940: 488), Hincks and Dibb (1958: 17).

**Comments.** This species is distributed in Bolivia, Brazil, Guiana, Suriname (Hincks and Dibb 1935, 1958; Fonseca and Reyes-Castillo 2004). Hincks (1940) recorded material from Bolivia as “Bolivia: Isiamas Dec. (W. M. Mann, Mulford Biol. Expl. 1921–1922)”.

**25. Passalus (Passalus) barrus Boucher & Reyes-Castillo, 1991**

Passalus (Passalus) barrus Boucher & Reyes-Castillo, 1991: Boucher and Reyes-Castillo (1991: 433).

**Material examined. Bolivia**: 6.VIII.1942. ex. Collection G. Griveau // PARATYPE (1 IEXA).

**Comments.** this species was described from Peru and Bolivia.

**26. Passalus (Passalus) coniferus Eschscholtz, 1829**

Passalus (Passalus) coniferus Eschscholtz, 1829: Hincks and Dibb (1935: 52).

**Material examined. Bolivia**: Dpto. de Cochabamba, Prov. Chapare, Sn. Antonio. IV-1953. Alt. 400 m. A. Martinez Col. // Selva tipo Amazónico (1 IEXA). Dpto. Cochabamba, Yungas del Palmar. III-1963. Alt. 2000 m. A Martínez col. // *Passalus coniferus* Eschscholtz. P. Reyes Castillo, det. 2005 (1 IEXA). Guanay. X.1989. sp65. M. Kon leg. 2004 (1 IEXA). Santa Cruz. Prov. Cordillera. Loc. Incahuasi. 16.III.2008 // Leg: Tito Vidaurre // Tipo de cebo Insectos (1 IEXA). Dpto. Santa Cruz, Prov. Florida, Samaipata, Abra de los Toros. 18 Nov. 2006. 18°7.113'S, 63°48.054'W. Altitud 2030 m. Bosque de lauráceas y helechos arborescentes. P. Reyes Castillo, col. // *Passalus coniferus* Eschscholtz. P. Reyes Castillo, det. 2008 (1 IEXA). Dpto. Santa Cruz, Prov. Florida, Samaipata, Paredones. 18 Nov 06. 18°8.437'S, 63°48.131'W. Altitud 1730m. Cultivo abandonado (chaco Viejo). P. Reyes Castillo, col. // *Passalus coniferus* Eschscholtz. P. Reyes Castillo, det. 2008 (4 IEXA). Dpto. Santa Cruz, Prov. Ichilo, Buenavista, III-951. Alt. 450 m. A Martinez, col. (2 IEXA). Santa Cruz. Ichilo, Buenavista. I-49. A Martínez, col. (1 IEXA). Santa Cruz. 4–6k SSE Buena Vista. F. & F. Hotel. 23–26 Oct. 2000. Wappes & Morris (1 IEXA). Santa Cruz. 4–6k SSE Buena Vista. F. & F. Hotel. Nov. 1–8 2002. J.E. Wappes (1 IEXA). Santa Cruz. Portachuelo. Sare. I-49 (2 IEXA).

**Comments.** Species with South American distribution: Argentina, Bolivia, Brazil, Colombia, Ecuador, Paraguay, Peru (Hincks and Dibb 1935, 1958; Amat-García et al. 2004). It was recorded erroneously from the Antilles (Jiménez-Ferbans et al. 2015).

**27. Passalus (Passalus) coarctatus Percheron, 1835**

Passalus (Passalus) coarctatus Percheron, 1835: Jiménez-Ferbans et al. (2016: 171).

**Material examined. Bolivia**: Beni; VII-26-VIII-4-1960; leg. B. Malkin// Chacobo Indian Village on Rio Benicito 66°–12°20' // *Passalus* (*P.) coniferus* Eschsch. Det: J. Schuster 2001 // Passalus (Passalus) coarctatus Percheron Det.: Jiménez-Ferbans, 2015 (1 FMNH). Santa Cruz, 5 km SSE Buena Vista, Hotel Flora y Fauna, 11.II.2007, CW LB O’Brien (1 UVGC).

**Comments.** Described from Brazil, *P. coarctatus* was then recorded from Bolivia, Brazil, Trinidad and Tobago, and Venezuela by Jiménez-Ferbans et al. (2016).

**28. Passalus (Passalus) inca Zang, 1905**

**Material examined. Bolivia**: Cochabamba, Yungas del Palmar. Alt. 2000 m. A. Martínez. Col. // Passalus (Passalus) inca Zang. Reyes-Castillo, det 85 (1 IEXA).

**Comments.**Zang (1905) described this species from “Peru: Chanchamayo”. This is the first record since the original description and first record from Bolivia.

**29. Passalus (Passalus) interruptus (Linneo, 1758)**

Passalus (Passalus) interruptus (Linneo, 1758): Hincks and Dibb (1935: 57).

**Material examined. Bolivia**: Dpto. Cochabamba, Prov. Chapare, Sn. Antonio. IV-1953. Alt. 400 m. A. Martínez col. Selva Amazónica (8 IEXA). Dpto. Cochabamba, El Palmar (Chapare), III-1953. Alt. 1000 m. A. Martínez col. // Bosque mixto de altura V. Amazónico (4 IEXA). Guanay. 21.VIII.1989. sp63. M. Kon leg. 2004 // Passalus (Passalus) interruptus (Linneo) Reyes-Castillo, det. 2005 (1 IEXA). Santa Cruz. Chiquitos, Santiago de Chiquitos-Río Tucavaca. 18°18'45.2"S, 59°33'0.4"W. 16-XI-2008. Alt. 319 m. // Bosque seco chiquitano. Bajo corteza de árbol pequi. W.D. Edmonds, P. Reyes, T. Vidaurre, col. // Passalus (Passalus) interruptus (Linnaeus, 1758) Reyes-Castillo, det. 2010 (5 IEXA). Dpto. Santa Cruz, Prov. Cordillera Parapeti. Dic. 1960. A. Martínez col. Bosque tropical caducifolio (1 IEXA). Santa Cruz. Ichilo, Buenavista, I-49. A Martínez, col. (2 IEXA). Depto. de Santa Cruz, Prov. Ichilo, Buenavista, III-49. Alt. 450 m. A Martínez, col. (1 IEXA). Dpto. Santa Cruz, Prov. Santa Cruz de la Sierra, Jardín Botánico. 7 noviembre 2006. W.D. Edmonds, col. (1 IEXA). Santa Cruz. Portachuelo. Sare. I-49 (1 IEXA). Dpto. Santa Cruz, Prov. Sara, Santa Rosa. XI-69. A. Martínez Col. (5 IEXA).

**Comments.** This species is distributed in South America and Panama (Reyes-Castillo and Castillo 1992).

**30. Passalus (Passalus) interstitialis Eschschltz, 1829**

Passalus (Passalus) interstitialis Eschschltz, 1829: Hincks and Dibb (1935: 58).

**Material examined. Bolivia**: Dpto. de Beni, Rurrenabaque erea. I-2006. Alt. 230 m. M. Kon, col. (2 IEXA). Depto. Cochabamba, Chapare, El Palmar. III-1953. Alt. 1000 m. A. Martinez Col. // Bosque mixto de altura V. Amazónico // Passalus (Passalus) interstitialis Eschscholtz, 1829. Reyes-Castillo, det. 2005 (3 IEXA). Dpto. Cochabamba, El Palmar (Chapare), III-1953. Alt. 1000 m. A. Martínez Col. // Bosque mixto de altura V. Amazónico // Passalus (Passalus) interstitialis Eschscholtz, 1829. Reyes-Castillo, det. 2005 (6 IEXA). Dpto. de Cochabamba, Prov. Chapare, Sn. Antonio. IV-1953. Alt. 400 m. A. Martinez col. // Selva tipo Amazónico (16 IEXA). Same data // Bosque tipo amazónico (11 IEXA). Guanay. Sp67. 19-VII-1989. M. Kon leg. 2004. (1 IEXA). Guanay. Sp60. XI-1989. M. Kon leg. 2004. (1 IEXA). Dpto. Santa Cruz. Provincia Chiquitos, Santiago de Chiquitos-Río Tucavaca. 18-diciembre-2008. Alt. 39 m. // 18°18'45.2"S, 59°33'0.4"W. W.D. Edmonds, P. Reyes, T. Vidaurre, col. Bajo corteza, tronco árbol de toboroche // Passalus (Passalus) interstitialis Eschscholtz, 1829. Reyes-Castillo, det. 2010 (26 IEXA). Santa Cruz: Chiquitos, Santiago de Chiquitos-Río Tucavaca. 16-XI-2008. Alt. 319 m. // 18°18'45.2"S, 59°33'0.4"W// Bosque seco chiquitano, bajo corteza de árbol de Pequi. W.D. Edmonds, P. Reyes, T. Vidaurre, col. // Passalus (Passalus) interstitialis Eschscholtz, 1829. Reyes-Castillo, det. 2010 (3 IEXA). Dpto. Santa Cruz, Provincia Cordillera Parapeti. Diciembre, 1960. A. Martínez col. // Bosque tropical caducifolio (1 IEXA). Dpto. Santa Cruz: Prov. Ichilo, Buena Vista. 16 noviembre 2006. Alt. 410 m. P. Reyes, col. // Passalus (Passalus) interstitialis Eschscholtz, 1829. Reyes-Castillo, det. 2008 (1 IEXA). Santa Cruz. 4–6k SSE Buena Vista. F. & F. Hotel. Nov. 2–12 Feb. 2000. J.E. Wappes // transition tropical forest 420–450 m (1 IEXA). Dpto. Santa Cruz. Prov. Ichilo. Buenavista (Tacú). 6-III-951. A Martínez, col. (1 IEXA). Santa Cruz. Reserva Natural Potrerillo del Guenda. 6–9 Oct. 2006. Wappes, Nearns & Eya // Snake Farm. 17°40.26'S, 63°27.43'W. Elevation 400 m (1 IEXA). Same data 16–22 Oct. 2006 (1 IEXA). Santa Cruz. Portachuelo. Sare. I-49 (23 IEXA). Dpto. Santa Cruz, Prov. Sara, Santa Rosa. XI-69. A. Martínez col. (10 IEXA). Sta. Cruz, Sierra, Sn. Miguel. 63°34'W, 17°27'S. VIII.77. Y. Camberfort, leg. // Passalus (Passalus) interstitialis Eschscholtz. Reyes-Castillo, det. 80 (1 IEXA). Santa Cruz. Rd. To Amboro above Achira. 14–15 Oct. 2006. Wappes, Nearns & Eya // Ag cut/burn area 18°07.43'S, 63°47.98'W. Elevation 1940 m (1 IEXA).

**Comments**. This is a common species distributed from Mexico to Argentina.

**31. Passalus (Passalus) opacus Gravely (1918)**

Passalus (Passalus) opacus Gravely (1918): Gravely (1918: 63), Hincks (1933: 179), Hincks and Dibb (1935: 60), Doesburg (1942: 334).

**Comments.** This species was described from a single specimen from “Farinas, Bolivia” (Gravely 1918). Hincks (1933) studied two specimens from “Coroico, Bolivia”.

**32. Passalus (Passalus) pugionatus Burmeister**

Passalus (Passalus) pugionatus Burmeister: Hincks (1940: 490), Hincks and Dibb (1958: 18).

**Comments.** described from Colombia, Hincks (1940) cited specimens from Bolivia, Colombia, Peru, and Venezuela. The specimens from Bolivia are referenced as “Coll. U.S.N.M.: Bolivia, Tumupasa Dec. (W. M. Mann, Mulford Biol. Expl. 1921–22)”.

**33. Passalus (Passalus) pugionifer Kuwert, 1891**

Passalus (Passalus) pugionifer Kuwert, 1891 Hincks (1933: 179, 1940: 488), Hincks and Dibb (1935: 56), Doesburg (1942: 333).

**Comments.** Originally described from Peru; Hincks (1933) cited “several specimens from Coroico, Bolivia”.

**34. Passalus (Passalus) punctiger Lepeletier & Serville, 1825**

Passalus (Passalus) punctiger Lepeletier & Serville, 1825: Hincks and Dibb (1935: 60).

**Material examined. Bolivia**: Dpto. Cochabamba, Chapare, El Palmar. III-1953. Alt. 1000 m. A. Martínez Col. // Bosque mixto de altura V. Amazónico // Passalus (Passalus) interstitialis Eschscholtz, 1829. Reyes-Castillo, det. 2005 (3 IEXA). Guanay. X.1992. sp52. M. Kon leg. 2004 // Passalus (Passalus) punctiger Lepeletier & Serville, 1825. Reyes-Castillo, det. 2005 (1 IEXA). Santa Cruz: Chiquitos, Santiago de Chiquitos-Río Tucavaca. 18°20'19.2"S, 59°35'9.7"W. 15-XI-2008. Alt. 725 m. // Bosque en galería. En parte húmeda y dura de tocón. Pareja en galería inicial. P. Reyes col. // Passalus (Passalus) punctiger Lepeletier & Serville, 1825. Reyes-Castillo, det. 2010 (1 IEXA). Santa Cruz: Chiquitos, Santiago de Chiquitos-Río Tucavaca. 18°19'6.8"S, 59°34'36.5"W. 14-XI-2008. Alt. 706 m. // Bosque seco chiquitano. En galería de tronco podrido de paquio Ficus sp. P. Reyes col. // Passalus (Passalus) punctiger Lepeletier & Serville, 1825. Reyes-Castillo, det. 2010 (1 IEXA). Santa Cruz: Chiquitos, Santiago de Chiquitos-Río Tucavaca. 18°16'9.7"S, 59°31'0.7"W. 19-XI-2008. Alt. 360 m. // Bosque seco chiquitano. En galería inicial de tronco delgado. P. Reyes col. // Passalus (Passalus) punctiger Lepeletier & Serville, 1825. Reyes-Castillo, det. 2010 (2 IEXA). Santa Cruz: Chiquitos, Santiago de Chiquitos-Río Tucavaca. 18°16'9.7"S, 59°31'0.7"W. 16-XI-2008. Alt. 360 m. // Bosque seco chiquitano. Bajo corteza. W.D. Edmonds, P. Reyes, T. Vidaurre, col. // Passalus (Passalus) punctiger Lepeletier & Serville, 1825. Reyes-Castillo, det. 2010 (1 IEXA). Same data // sp55 (1 IEXA). Same data // sp62 (1 IEXA).

**Comments.** This is a common species distributed from Mexico to Argentina.

**35. Passalus (Passalus) unicornis Lepeletier & Serville, 1825**

Passalus (Passalus) unicornis Lepeletier & Serville, 1825 Luederwaldt (1931b: 188), Hincks and Dibb (1935: 63).

**Comments.** Described from Cayenne, French Guiana, this species has been recorded from the Lesser Antilles, Bolivia, Brazil, and Colombia. Jiménez-Ferbans et al. (2013) considered the citation from Argentina as dubious. Similarly, we consider the record from Guatemala by Hincks and Dibb (1935) as dubious. Luederwaldt (1931b) cited an exemplar from “Bolivia, Steinbach leg., immature”, remarking that it only has 29 mm total length. We doubt that this specimen belongs to *P. unicornis*, a species with a total length of 36–45 mm (Jiménez-Ferbans et al. 2016).

### Descriptions of new species

#### Passalus (Pertinax) bolivianus sp. nov.

Taxon classificationAnimaliaColeopteraScarabaeoidea

CAAAE765-2E47-5294-BBDE-4D33A173226D

http://zoobank.org/E6304B84-71B7-481C-A525-CB6B2B4692AE

Figs 1–4

##### Material examined.

Holotype: female, pinned, BOLIVIA: COCHABAMBA, Prov. Carrasco, Yungas. ii.1971. alt. 3200 m. A. Martínez col. // Bosque húmedo de montaña de *Podocarpus* (CEBUMAG-ENT). Paratypes: 2 males, 8 females, 18 unsexed, same data as holotype (IEXA, FMNH). 1 female, BOLIVIA: COCHABAMBA, Prov. Carrasco, Serranía de Siberia, Chua Khocha // 30.viii.1990, No. 093, cloud forest, 2300 m inside log, M. Ledezma Field Museum // #93 // Passalus (Pertinax) n. sp. det.: Jiménez-Ferbans 2015 // Ilustrado por Rivera-Gasperin (FMNH). 1 specimen, BOLIVIA: SANTA CRUZ, Florida, 4km S. De Samaipata 1800 m alt., 7.xii. 1991, B.N. Smith (IEXA).

**Figures 1–4.**
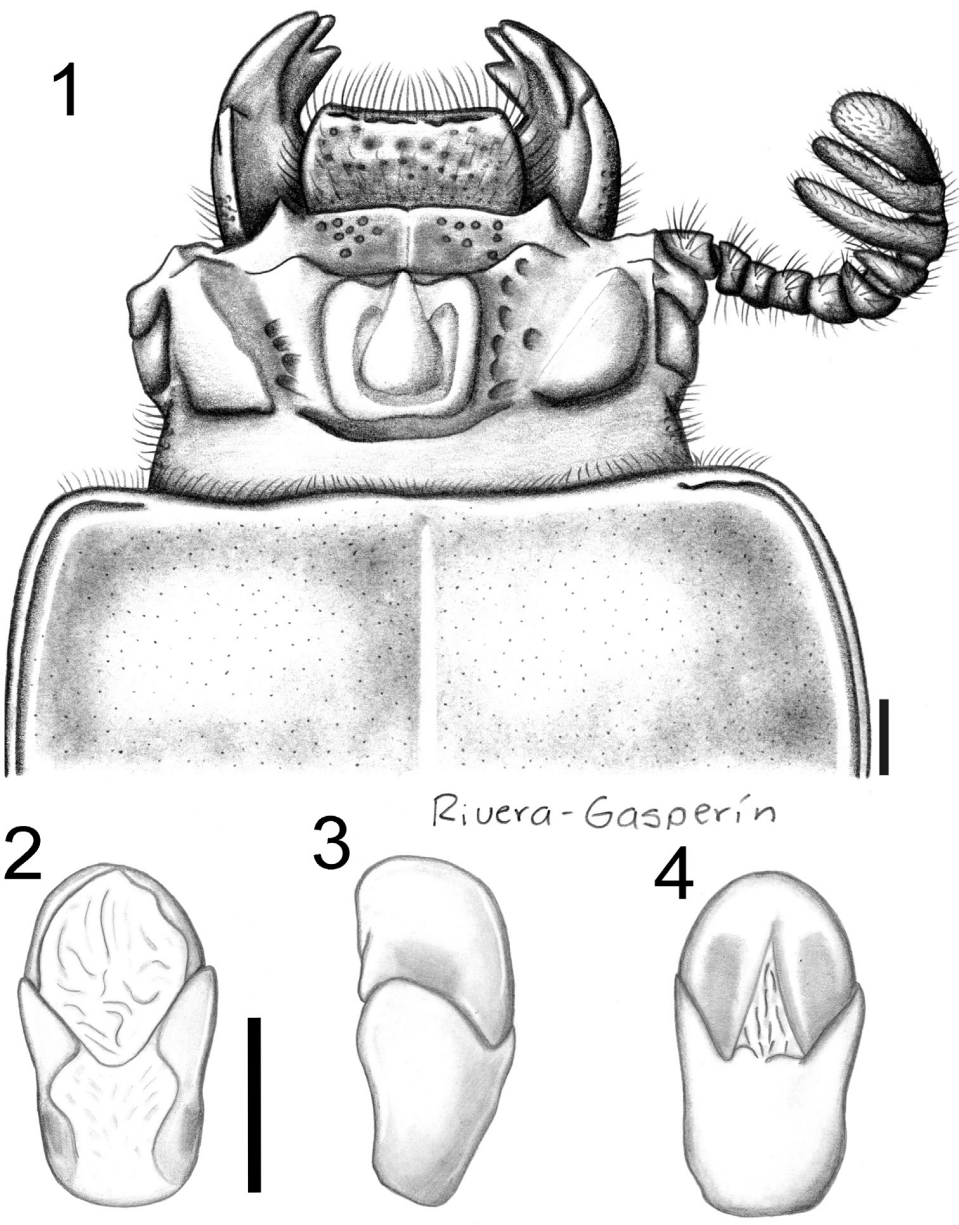
Passalus (Pertinax) bolivianus sp. nov. **1** dorsal view of the head and anterior part of pronotum **2–4** Aedeagus **2** dorsal view **3** lateral view **4** ventral view. Scale bars: 1 mm.

##### Diagnosis.

Passalus (Pertinax) bolivianus sp. nov. differs from other brachypterous species of Passalus (Pertinax) by having lateroposterior tubercles larger that central tubercle, anterior border of frons almost straight with small middle indentation, rounded punctures on both lateral and dorsal elytral striae, and elytral humeri heavily pubescent.

##### Description.

***Habitus***: midsize, total length 32.8–36.8 mm, brachypterous, body convex, shiny, black.

***Head***: labrum with anterior border straight or slightly concave, covered with setae that are less dense anteriorly. Clypeus hidden under the frons, with anterior angles reduced under the mediofrontal tubercles and smaller than mediofrontal tubercles. Frons narrow, anterior frontal edge with small middle indentation, without secondary mediofrontal tubercles. Mediofrontal tubercles projected forward, larger than internal tubercles. Internal tubercles small, conical, with apex not free, joined to mediofrontal tubercles by a weak ridge, located midway between mediofrontal tubercles and central tubercle apex. Posterofrontal ridges V-shaped. Area between the frontal ridges with scarce punctures on the anterior half, divided by a longitudinal sulcus running from border of frons to the base of central tubercle. Cephalic tumescence (= mamelon sensu Jiménez-Ferbans and Reyes-Castillo 2014) divided. Mesofrontal structure of the “marginatus” type (Reyes-Castillo 1970), central tubercle wide at the base with a sulcus posteriorly, apex not free. Lateroposterior tubercles marked, conical and large, larger than central tubercle. Lateropostfrontal areas glabrous, shiny, and impunctate. Eyes reduced, canthus glabrous, covering ½ of the eye in lateral view. Postorbital pits weak. Postfrontal groove semicircular and complete, with small inverted v-shape in central part. Hypostomal process slightly separated from mentum, glabrous, extending anteriorly to the superior part of the middle zone of the mentum. Medial basal mentum protruding ventrally, laterally pubescent. Mentum with large lateral fossae that are shallow and pubescent. Antennal club trilamellate, lamellae elongate. Internal tooth of the left mandible bidentate, simple on right mandible. Dorsal tooth longitudinally straight in dorsal view but slightly sinuous in lateral view. Dorsal mandibular pubescence covering the base of mobile tooth. Mandibular fossae reaching base of mobile tooth. Maxilla with lacinia apically bidentate. Ligula tridentate, middle tooth longer than lateral teeth. Middle palpomere of the labial palp 1.3 times wider, and 1.1 times longer, than distal palpomere.

***Thorax***: Pronotum rounded in dorsal view, wider than elytra, with punctures restricted to areas around lateral fossae and marginal groove. Marginal groove narrow, clearly visible along anterior angles, extending along approximately 1/3 of the anterior margin of the pronotum; median longitudinal sulcus and lateral fossae well marked. Inferolateral area of pronotum with abundant pubescence. Prosternellum rhomboidal, opaque. Pre-epimeron (sensu Reyes-Castillo 1970) shiny and fully pubescent. Mesosternum with small, rounded, mesosternal scar, glabrous, lateral area opaque. Posterior corner of the mesepisternum and mesepimeron glabrous. Metasternum pubescent anteriorly and in lateral fossa; metasternal disc delimited by numerous punctures medially and posteriorly. Metasternal lateral fossa and epipleuron of similar width.

***Elytron***: Shiny, anterior border rounded and pubescent. Humerus and epipleuron pubescent. Rounded punctures on lateral and dorsal striae (but more strongly on lateral striae).

***Leg***: Femur I with ventral anterior marginal sulcus narrow and complete (reaching the apical pubescence). Tibia I with dorsal sulcus complete. Tibia II with one weak spine and tibia III unarmed.

***Abdomen***: Marginal groove of posterior-most sternite complete.

***Aedeagus***: Basal piece fused with parameres in ventral view (Fig. 4). Ventral surface of median lobe almost entirely sclerotized, measured along media ventral line, length of medial lobe 0.9 times that of basal piece and parameres. Lateral projections of parameres small and apices rounded in lateral view (Fig. 3).

##### Etymology.

Named after the country, Bolivia.

##### Variations.

The anterior border of the labrum can be straight or slightly concave. The longitudinal sulcus on the area between frontal ridges can be weak or marked. Medial basal mentum can be fully pubescent or only laterally so.

##### Taxonomic discussion.

Passalus (Pertinax) bolivianus sp. nov. is similar in size and habitus to *Passalus nudifrons* Dibb, from which it differs by having anterior border of head straight with central excision, humeri pubescent and anterior area of metasternum punctate and pubescent. Likewise, the total length of *P. bolivianus* sp. nov. is similar to that of *P. gonzalezae* sp. nov., but the former has elytral striae with rounded punctures, marked on both lateral and dorsal striae (weak punctures on striae 7–10 in *P. gonzalezae*) and humeri heavily pubescent.

#### Passalus (Pertinax) gonzalezae sp. nov.

Taxon classificationAnimaliaColeopteraScarabaeoidea

5B8CFE06-F046-58BF-82C0-7B53FCCB9DCE

http://zoobank.org/BF2BA672-2764-4F13-8021-A0B9C4F04988

Fig. 5

##### Material examined.

Holotype: female, pinned, BOLIVIA: Yungas, Incachaca, 2800 m, xii.1960, Zischka leg. // Passalus (Pertinax) n. sp. Det.: Jiménez-Ferbans, 2016.

##### Diagnosis.

Among the brachypterous species of Passalus (Pertinax), *P. gonzalezae* sp. nov. is recognizable by the absence of punctures on frontal area (delimited by the frontal ridges), by having anterior border of head with strong (deep) middle indentation, insinuating secondary mediofrontal tubercles, and weak punctures on elytral striae 7–10.

**Figure 5.**
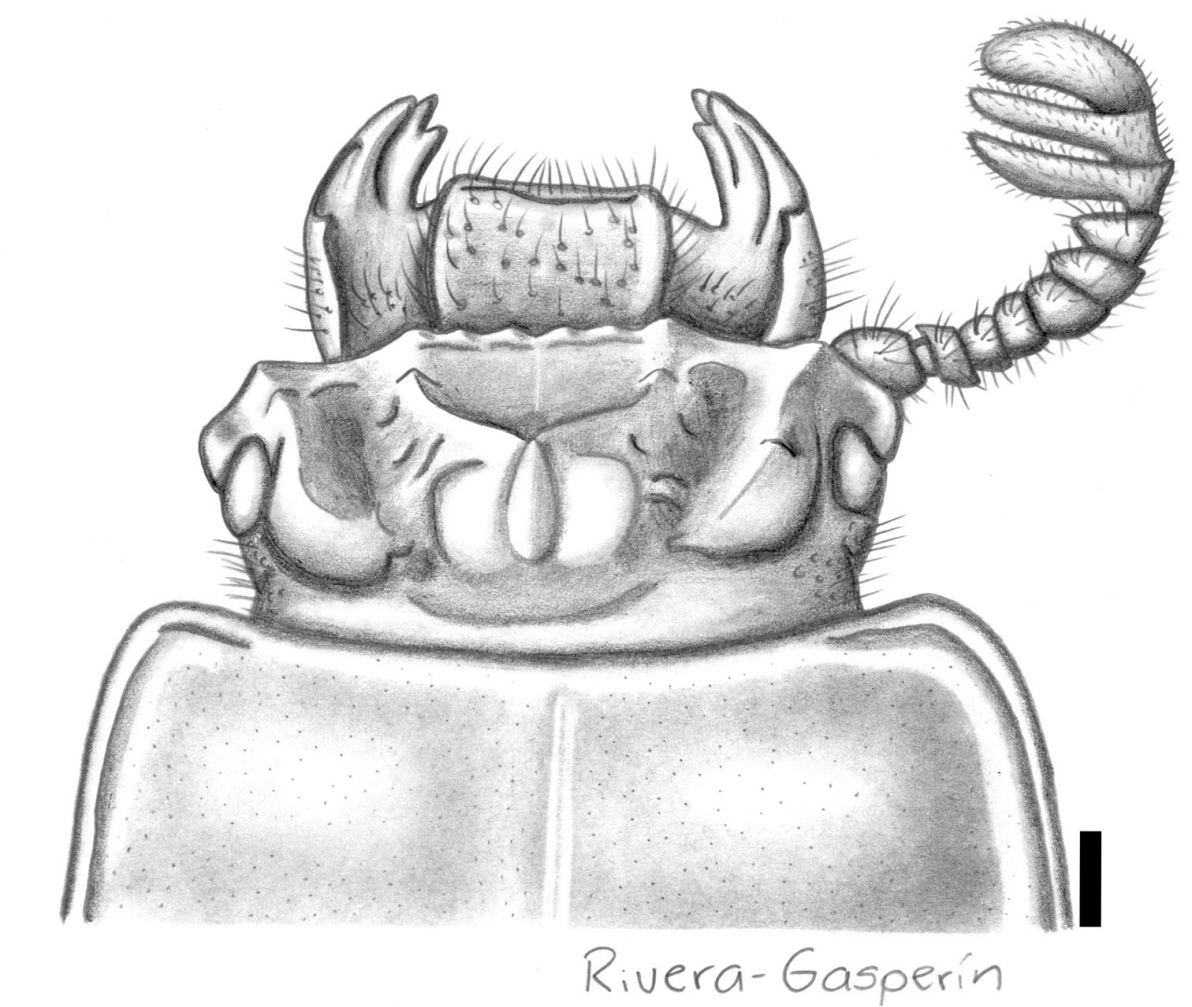
Passalus (Pertinax) gonzalezae sp. nov., dorsal view of the head and anterior part of pronotum. Scale bar: 1 mm.

##### Description.

***Habitus***: midsize, total length 31.3 mm, brachypterous, body convex, reddish (teneral).

***Head***: labrum with anterior border almost straight, covered with setae uniformly. Clypeus hidden under the frons, with anterior angles reduced under the mediofrontal tubercles and smaller than mediofrontal tubercles. Frons narrow, anterior frontal edge with strong median indentation, insinuating secondary mediofrontal tubercles. Mediofrontal tubercle projected anteriorly and similar in size to internal tubercle. Internal tubercle midway between mediofrontal tubercles and apex of central tubercle, apex not free, nor joined to mediofrontal tubercles by a ridge. Posterofrontal ridges V-shaped. Area between the frontal ridges without punctures, divided by a longitudinal sulcus from the border of frons to the base of cephalic tumescence (= mamelon sensu Jiménez-Ferbans and Reyes-Castillo 2014). Cephalic tumescence not divided. Mesofrontal structure of the “marginatus” type (Reyes-Castillo 1970), with central tubercle wide at the base, lacking posterior sulcus, apex not free. Lateroposterior tubercle marked but small, smaller than central tubercle. Lateropostfrontal area glabrous, shiny, and impunctate. Eye reduced, canthus covering 2/3 of eye in lateral view. Left canthus with two setae, right canthus glabrous. Postorbital pit weak. Postfrontal groove semicircular, complete and with small inverted v-shape in central part. Hypostomal process slightly separated from mentum, glabrous and extending anteriorly to superior part of the middle zone of the mentum. Medial basal mentum protruding ventrally, laterally pubescent. Mentum with large lateral fossae, shallow and pubescent. Antennal club tri-lamellate, with lamellae elongate. Internal tooth of left mandible bidentate, simple on right mandible. Dorsal tooth straight in dorsal view and slightly sinuous in lateral view. Dorsal mandibular pubescence covering base of mobile tooth. Mandibular fossae reaching base of mobile tooth. Lacinia apically bidentate. Ligula tridentate, middle tooth slightly longer than lateral teeth. Middle labial palpomere same width as, and 1.1 times longer than, distal palpomere.

***Thorax***: Pronotum rounded in dorsal view, wider than elytra, with 34 punctures on lateral fossae areas and three punctures restricted to the area of the marginal groove. Marginal groove narrow, visible at anterior angles and extending 1/3 length of anterior margin of pronotum. Longitudinal sulcus and lateral fossa well marked. Inferolateral area of pronotum with sparse pubescence. Prosternellum rhomboidal, shiny. Pre-epimeron (sensu Reyes-Castillo 1970) shiny and glabrous. Mesosternum with mesosternal scar oval, glabrous, lateral area opaque. Posterior corner of the mesepisternum and mesepimere glabrous. Anterolateral part of metasternum smooth and glabrous. Metasternum glabrous anteriorly and in lateral fossa; metasternal disc smooth (without punctures), delimited by numerous punctures posteriorly. Posterior metasternal lateral fossa less wide than epipleura.

***Elytron***: Shiny, anterior border rounded and glabrous. Humerus and epipleuron glabrous. Striae with rounded punctures, barely perceptible on striae 5–10.

***Leg***: Femur I with ventral anterior marginal sulcus narrow and complete, reaching the apical pubescence. Tibia I with dorsal sulcus complete. Tibia II with one weak spine and tibia III unarmed.

***Abdomen***: Marginal grove of posterior-most sternite complete.

**Etymology.** This species is named in honor of Dr. Dolores Gonzalez from Instituto de Ecología A.C. (Mexico), who has collaborated with the authors in molecular phylogenetic studies of Passalidae.

**Taxonomic discussion.***Passalus gonzalezae* sp. nov. is similar to *P. catharinae* Gravely, 1918 (31–33 mm) from which it differs by the absence of punctures on frontal area, by having anterior border of head with strong (deep) middle indentation, so strong that it produces the appearance of being flanked by secondary mediofrontal tubercles, apex of central tubercle not free (attached to the frons), the reduced wings, and weak punctures on striae 7–10. From other brachypterous species, *P. gonzalezae* sp. nov. is similar to *P. nudifrons* and *P. bolivianus* sp. nov. However, *P. nudifrons* has the head with anterior margin shallowly concave, without central excision, while in *P. gonzalezae* sp. nov. the anterior frontal edge has a strong median indentation, insinuating secondary mediofrontal tubercles. From *P. bolivianus* sp. nov., *P. gonzalezae* sp. nov. differs by having weak punctures on striae 7–10 (strong in *P. bolivianus* sp. nov.) and humeri glabrous.

#### Passalus (Pertinax) canoi sp. nov.

Taxon classificationAnimaliaColeopteraScarabaeoidea

888B5011-51AA-5777-A9E9-0B76220D2DE3

http://zoobank.org/3E5C476C-2106-4100-B3E1-9402E5EDDD65

Figs 6 , 7–9

##### Material examined.

Holotype: female, pinned, BOLIVIA: Yungas del Palmar, 15.iii.1958, 2000 m M. Zlsekka // “*Publius*” *spinipes* Zang Det.: JCS [Jack C. Schuster] ’95 [1995] // Passalus (Pertinax) sp. n. Reyes-Castillo det. 2013 (UVG). Paratype: female, pinned BOLIVIA: COCHABAMBA, Yungas del Palmar // iii.1963, Alt. 2000 m A. Martínez col. // Passalus (Pertinax) n. sp. det.: Jiménez-Ferbans 2015 // Ilustrado por Rivera-Gasperin (CEBUMAG-ENT)

**Figure 6.**
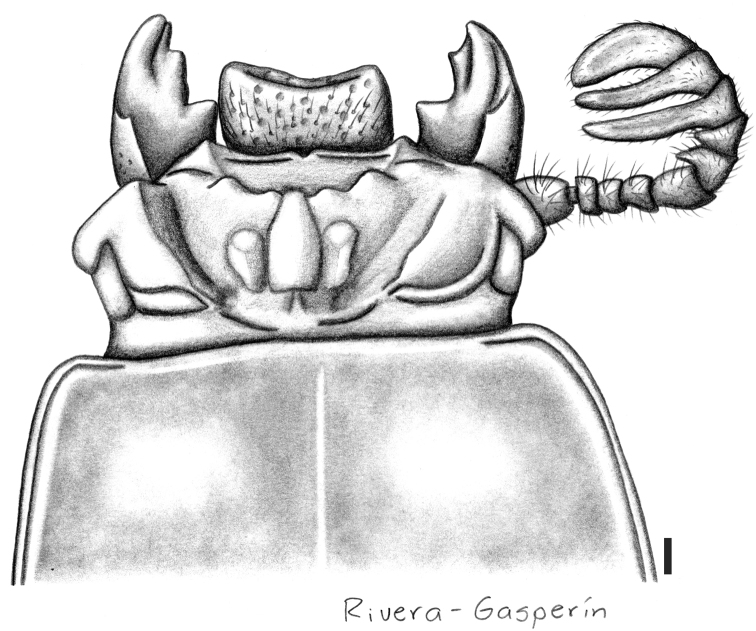
Passalus (Pertinax) canoi sp. nov., dorsal view of the head and anterior part of pronotum. Scale bar: 1 mm.

##### Diagnosis.

*P. canoi* sp. nov. is diagnosable by its large size (45.0–46.0 mm), strong indentation on frontal edge, internal tubercles joined to medifrontal tubercles by a weak ridge, humeri and epipleura glabrous, inferolateral area of pronotum with sparse pubescence, and metasternal disc delimited by punctures only posteriorly.

##### Description.

***Habitus***: large size, total length 45.2–46.0 mm, brachypterous, body convex, shiny, black.

***Head***: labrum with anterior border concave, covered with setae that are less dense in anterior border. Clypeus hidden under the frons, anterior angles reduced under mediofrontal tubercles and smaller than mediofrontal tubercles. Frons narrow, anterior frontal edge with strong middle indentation, insinuating secondary mediofrontal tubercles. Mediofrontal tubercle projected forward, larger than internal tubercle. Internal tubercle located midway between mediofrontal tubercles and the central tubercle apex, apex not free, weakly joined to mediofrontal tubercles by a weak ridge. Posterofrontal ridges V-shaped. Area between the frontal ridges lacking punctures. Cephalic tumescence (= mamelon sensu Jiménez-Ferbans and Reyes-Castillo 2014) not divided. Mesofrontal structure of the “marginatus” type (Reyes-Castillo 1970), with central tubercle wide at the base, lacking posterior sulcus, apex not free. Lateroposterior tubercle large. Lateropostfrontal area glabrous, shiny, and impunctate. Eye reduced, canthus covering 3/4 of eye in lateral view. Canthus glabrous. Postorbital pit weak. Postfrontal groove semicircular and complete, with small inverted v-shape in central part. Hypostomal process slightly separated from mentum, glabrous and extending anteriorly to the superior part of the middle zone of the mentum. Medial basal mentum protruding ventrally, glabrous. Mentum with large lateral fossae, shallow and pubescent (the fossae is glabrous). Antennal club tri-lamellate, with lamellae elongate. Internal tooth of left mandible bidentate, simple on right mandible. Dorsal tooth straight in dorsal view and slightly concave in lateral view. Dorsal mandibular pubescence covering base of mobile tooth. Mandibular fossae reaching base of mobile tooth. Lacinia apically bidentate. Ligula tridentate, middle tooth longer than lateral teeth. Middle labial palpomere same length as, and 1.5 times wider than, distal palpomere.

***Thorax***: Pronotum rounded, wider than elytra, with punctures restricted to lateral fossae (12 on right and 14 on left). Marginal groove narrow, visible in anterior angles, and extending along 1/3 of anterior margin of pronotum; longitudinal sulcus well marked. Lateral fossae marked. Inferolateral area of pronotum with sparse pubescence. Prosternellum rhomboidal, opaque. Pre-epimeron (sensu Reyes-Castillo 1970) shiny and fully pubescent. Mesosternum with mesosternal scar small and rounded, glabrous; lateral area opaque. Posterior corner of mesepisternum and mesepimeron glabrous. Anterolateral part of metasternum smooth and glabrous. Anterior portion and lateral fossa of metasternum glabrous; metasternal disc delimited by punctures posteriorly; metasternal lateral fossa narrower than epipleura.

***Elytron***: Shiny, anterior border rounded and glabrous. Humerus and epipleuron glabrous. Striae with rounded punctures (weak), stronger on lateral striae than on dorsal striae.

***Leg***: Femur I with ventral anterior marginal sulcus narrow and complete (reaching the apical pubescence). Tibia I with dorsal sulcus complete. Tibia II and III with one weak spine.

***Abdomen***: Marginal groove of posterior-most sternite complete.

##### Etymology.

This species is named in honor of Dr. Enio Cano from Guatemala, a passionate scholar of Scarabaeoidea.

##### Variation.

Five punctures on the anterior half (paratype), punctations restricted to the lateral fossae (11 on right and 82 on the left).

##### Taxonomic discussion.

The size of *P. canoi* sp. nov. easily differentiates this species from other brachypterous Passalus (Pertinax). However, the habitus and strong indentation on frontal edge can make it similar to *P. gonzalezae* sp. nov., from which *P. canoi* sp. nov. differs by having a weak ridge joining the internal tubercles with mediofrontal tubercles; this characteristic also makes *P. canoi* sp. nov. different from *P. nudifrons*. Another difference is the medial basal mentum glabrous in *P. canoi* sp. nov. and laterally pubescent in *P. gonzalezae* sp. nov., and the frontal area divided by a longitudinal sulcus from the border of frons to the base of cephalic tumescence in *P. gonzalezae* sp. nov. (there is no sulcus in *P. canoi* sp. nov.).

**Figures 7–9.**
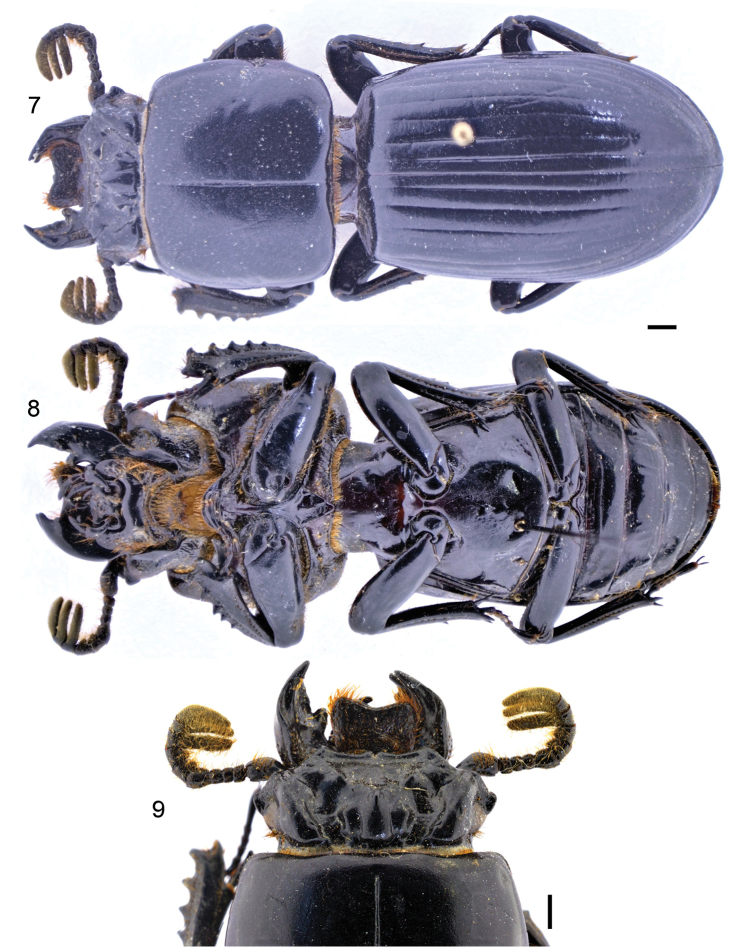
Passalus (Pertinax) canoi sp. nov. **7** dorsal habitus **8** ventral habitus **9** dorsal view of the head and anterior part of pronotum. Scale bars: 2 mm.

### Key to the Passalidae from Bolivia

Since the fauna of Passalidae from Bolivia is still poorly known, this key must be used with caution. It is probable that future surveys will yield new species and new country; for this reason, it is convenient to use this key and then confirm the determination with original description or diagnosis of the species.

**Table:** 

1	Clypeus hidden below frons, with anterior angles below mediofrontal tubercles (Fig. 11)	**2**
–	Clypeus (frontoclypeus) exposed dorsally, with anterior angles in front of border of frons (Fig. 10)	**28**
2	Maxilla with lacinia unidentate or bidentate in apical third (Fig. 14). Antennal club with five lamellae (Fig. 18). Mediobasal area of mentum flat (Fig. 12). Prosternellum pentagonal (*Paxillus*) (Fig. 16)	**3**
–	Maxilla with lacinia bidentate in apical third (Fig. 15). Antennal club with three lamellae (five in *Passalus rhodocanthopoides* and four in *P. interstitialis*) (Figs 19–21). Mediobasal area of mentum protruding (Fig. 13). Prosternellum rhomboidal (Fig. 17) (*Passalus*)	**7**
3	Maxilla with lacinia unidentate in apical third (Fig. 14). Anterior border of frons straight, without secondary mediofrontal tubercles (Fig. 11)	**4**
–	Maxilla with lacinia bidentate in apical third (Fig. 15). Anterior border of frons with two small secondary mediofrontal tubercles (Fig. 18)	**5**
4	Dorsal mandibular tooth with a concave expansion (in dorsal view). Mesosternum smooth, without punctures over mesosternal scar. Body length 16.0–19.5 mm	***Paxillus leachi* MacLeay**
–	Dorsal mandibular tooth thin, without a concave expansion. Mesosternum with punctures over mesosternal scar and beyond. Body length 14.0–16.0 mm	***Paxillus camerani* (Rosmini)**
5	Mesosternal scar oval, weakly defined, shiny. Metasternal fossae and epipleura glabrous. Body length 18.0–19.0 mm	***Paxillus forsteri* Luederwaldt**
–	Mesosternal scar elongate, well-defined, and rugose. Metasternal fossae and epipleura pubescent	**6**
6	First lamella of antennal club reduced. Body length 22.7–23.1 mm (Fig. 18)	***Paxillus martinezi* Jiménez-Ferbans & Reyes-Castillo**
–	First lamella of antennal club not reduced, almost equal in width to second lamella. Body length 18.5–19.5 mm	***Paxillus pleuralis* Luederwaldt**
7	Anterior border of frons straight or almost straight, without secondary mediofrontal tubercles (Fig. 19). Central tubercle with apex not free, fused with frontal ridges (subgenus Pertinax)	**8**
–	Anterior border of frons with one or two secondary mediofrontal tubercles (Figs 20–21); if not, and border is straight, then central tubercle with apex distinctly free (reaching or almost reaching frons border)	**15**
8	Antennal club with 5 lamellae, first two reduced (half width of third lamella). Body length 22.0–25.0 mm	**Passalus (Pertinax) rhodocanthopoides**
–	Antennal club with three lamellae (Fig. 19)	**9**
9	Wings reduced (brachypterous) (Fig. 7)	**10**
–	Wings fully developed (macropterous)	**13**
10	Lateroposterior tubercles larger that central tubercle (Fig. 1). Elytral humeri heavily pubescent. Body length 32.8–36.8 mm	**Passalus (Pertinax) bolivianus sp. nov.**
–	Lateroposterior tubercles smaller that central tubercle (Figs 5–7). Elytral humeri glabrous	**11**
11	Internal tubercles joined to mediofrontal tubercles by a weak ridge. Frontal area, between frontal ridges, not divided by a longitudinal sulcus (Figs 6, 9). Medial basal mentum glabrous (Fig. 8). Body longer (45.0–46.0 mm)	**Passalus (Pertinax) canoi sp. nov.**
–	Internal tubercles not joined to mediofrontal tubercles by a ridge (Fig. 5). Frontal area divided by a longitudinal sulcus from border of frons to base of cephalic tumescence. Medial basal mentum laterally pubescent. Body shorter (31.0–32.0 mm)	**12**
12	Anterior frontal border with strong median indentation, insinuating secondary mediofrontal tubercles (Fig. 5). Body length 31.3 mm	**Passalus (Pertinax) gonzalezae sp. nov.**
–	Anterior frontal edge straight, without median indentation. Body length 32.0 mm	**Passalus (Pertinax) nodifrons Dibb**
13	Apex of central tubercle slightly free (the very tip not detached to the frontal ridgeds and frontal area). Body length 31.0–33.0 mm	**Passalus (Pertinax) catharinae Gravely**
–	Apex of central tubercle not free, fused with frontal ridges (Fig. 19)	**14**
14	Humeri with sparse pubescence at base. Body shorter (25.1–28.0 mm)	**Passalus (Pertinax) morio Percheron**
–	Humeri glabrous. Body longer (42.2–44.3 mm) (Fig. 19)	**Passalus (Pertinax) convexus Dalman**
15	Anterior border of frons with one secondary mediofrontal tubercle. Central tubercle with apex not free. Hypostomal process with a matt groove over apex	**Passalus (Mitrorhinus) lunaris (Kaup)**
–	Anterior border of frons with two secondary mediofrontal tubercles; if border straight, then central tubercle with apex distinctly free (“Petrejus” group). Hypostomal process without a matt groove over apex (subgenus Passalus)	**16**
16	Anterior border of frons with two secondary mediofrontal tubercles joined at bases	**17**
–	Anterior border of frons with or without mediofrontal tubercles, when present secondary mediofrontal tubercles separated	**18**
17	Secondary mediofrontal tubercles large and fused with each other almost totally. Lateropostfrontal area glabrous. Body length 24.3–27.0 mm	**Passalus (Passalus) barrus Boucher & Reyes-Castillo**
–	Secondary mediofrontal tubercles only contiguous at base. Lateropostfrontal area pubescent. Body length 31.1–33.0 mm	**Passalus (Passalus) abortivus Percheron**
18	Central tubercle with apex very free, reaching or surpassing anterior border of frons. Secondary mediofrontal tubercles absent or rudimentary (“Petrejus” group)	**19**
–	Central tubercle with apex not free or slightly free (Fig. 21); if reaching anterior border of frons, then metasternum densely pubescent (anterior and lateral areas). Secondary mediofrontal tubercles always present and large (Figs 20–21) (“Neleus” group)	**22**
19	Central tubercle surpassing widely anterior margin of head, fused to median portion of head almost to anterior margin. Body length 24.0 mm	**Passalus (Passalus) pugionifer Kuwert**
–	Central tubercle not fused to median portion of head	**20**
20	Central tubercle concave at apex. Body longer (40.0–51.0 mm)	**Passalus (Passalus) armatus Perty**
–	Central tubercle acute, not concave at apex. Body shorter (23.0–30.0 mm)	**21**
21	Central tubercle strongly sulcate at base. Humeri pubescent. Body length 30.0 mm	**Passalus (Passalus) inca Zang**
–	Central tubercle not sulcate at base. Humeri glabrous. Body length 23.0–30.0 mm	**Passalus (Passalus) pugionatus Burmeister**
22	Habitus opaque. Body length 39.5 mm	**Passalus (Passalus) opacus Gravely**
–	Habitus shiny	**23**
23	Mesosternal fossae glabrous or with only 1–3 setae (*P. interruptus*)	**24**
–	Mesosternal fossae densely pubescent	**27**
24	Antennal club with four lamellae, fourth one very reduced and tomentose. Body length 27.1–34.0 mm	**Passalus (Passalus) interstitialis Eschscholtz**
–	Antennal club with three lamellae	**25**
25	Central tubercle very free, reaching anterior border of head. Pronotum pubescent on lateral fossae. Body length 36.0–45.1 mm	**Passalus (Passalus) unicornis Lepeletier & Serville**
–	Central tubercle slightly free, not reaching anterior border of head. Lateral fossa of pronotum glabrous	**26**
26	Last abdominal sternite with incomplete groove. Body longer (44.4–52.8 mm) (Fig. 20)	**Passalus (Passalus) interruptus (Linneo)**
–	Last abdominal sternite with medially complete groove. Body shorter (29.1–42.0 mm) (Fig. 21)	**Passalus (Passalus) punctiger Lepeletier & Serville**
27	Central tubercle with apex very free, reaching anterior cephalic border. Body length 33.0–38.0 mm	**Passalus (Passalus) coarctatus Percheron**
_	Central tubercle with apex not free or barely free. Body length 34.2–39.1 mm	**Passalus (Passalus) coniferus Eschscholtz**
28	Frontoclypeal suture present	**29**
–	Frontoclypeal suture absent	**30**
29	Antennal club with three lamellae. Body length 18.2–23.1 mm	***Popilius marginatus* (Percheron)**
–	Antennal club with four or five lamellae. Body length 17.0–21.0 mm	***Popilius tetraphyllus* (Eschscholtz)**
30	Anterior labral border deeply concave, with an excavation behind concavity (dorsal depression sensu Marshall 2000). Body length 38.5–40.2 mm	***Verres furcilabris* (Eschschltz)**
–	Anterior labral border straight or slightly concave or convex, without an excavation behind border (*Veturius*)	**31**
31	Brachypterous. Body length 34.0–45.0 mm	**Veturius (Publius) spinipes (Zang)**
–	Macropterous (subgenus Veturius)	**32**
32	Mesosternum glabrous (not including anterior angles, which can have some scarce short setae)	**33**
–	Mesosternum with dense pubescence	**36**
33	Central tubercle with apex free. Body length 36.0–40.0 mm	**Veturius (Veturius) libericornis Kuwert**
–	Central tubercle with apex not free	**34**
34	Lateropostfrontal areas pubescent (rarely glabrous). Metasternum with pubescence beyond anterior border (mesocoxal cavity) and lateral fossa, reaching lateromedial metasternum. Body length 37.0–49.0 mm	**Veturius (Veturius) standfussi Kuwert**
–	Lateropostfrontal areas glabrous. Metasternum with pubescence restricted to anterior border (mesocoxal cavity) and lateral fossa	**35**
35	Postfrontal groove (occipital sulcus sensu Reyes-Castillo 1970) absent. Superior spurs of mesotibiae and metatibiae curved. Body length 39.0–43.0 mm	**Veturius (Veturius) guntheri Kuwert**
–	Postfrontal groove present. Superior spurs of mesotibiae and metatibiae straight or almost straight. Body length 39.0–46.0 mm	**Veturius (Veturius) yahua Boucher**
36	Lateropostfrontal area glabrous. Body length 33.0–41.0 mm	**Veturius (Veturius) sinuosus (Drapiez)**
–	Lateropostfrontal area pubescent	**37**
37	Lateropostfrontal area with 2–15 long setae. Central tubercle high, in lateral view higher than internal tubercles. Body length 30.0–37.0 mm	**Veturius (Veturius) boliviae Gravely**
–	Lateropostfrontal area with 2–10 short setae. Central tubercle almost at same level of internal tubercles in lateral view. Body length 28.0–30.0 mm	**Veturius (Veturius) dreuxi Boucher**

**Figures 10–18.**
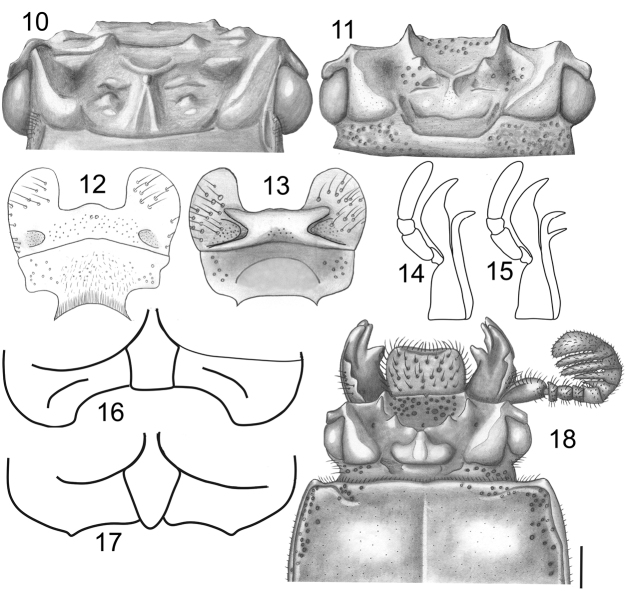
**10, 11** Head: **10***Veturius* sp. **11***Paxillus* sp. **12, 13** mentum, ventral view: **12***Paxillus leachi***13***Passalus* sp. **14, 15** ventral view of right maxilla: **14***Paxillus***15***Passalus***16, 17** Prosternelum: **16***Paxillus***17***Passalus***18** head and anterior part of pronotum of *Paxillus martinezi*. Scale bar: 1 mm.

### Clave para las especies de Passalidae de Bolivia

Dado que la fauna de Passalidae de Bolivia aún es poco conocida, esta clave debe usarse con precaución. Es probable que estudios futuros encuentren nuevas especies y registros para el país; por ese motivo, es conveniente utilizar esta clave y luego confirmar la determinación con la descripción original o el diagnóstico de la especie.

**Table:** 

1	Clípeo oculto debajo de la frente, con ángulos anteriores debajo de los tubérculos mediofrontales (Fig. 11)	**2**
–	Clípeo (clípeo-frente) expuesto dorsalmente, con ángulos anteriores por delante del borde frontal (Fig. 11)	**28**
2	Maxila con lacinia uni o bidentada en el tercio apical (Fig. 14). Maza antenal con cinco lamelas (Fig. 18). Parte mediobasal del mentón plana (Fig. 12). Presternelo pentagonal (*Paxillus*) (Fig. 16)	**3**
–	Maxilla con lacinia bidentada en el tercio apical (Fig. 15). Maza antenal con tres lamelas (cinco en *Passalus rhodocanthopoides* y cuatro en *P. interstitialis*) (Figs 19–21). Parte mediobasal del mentón abultada (Fig. 13). Presternelo romboidal (Fig. 17) (*Passalus*)	**7**
3	Maxilla con lacinia bidentada en el tercio apical (Fig. 14). Borde anterior de la frente recto, sin tubérculos mediofrontales secundarios (Fig. 11)	**4**
–	Maxilla con lacinia unidentada en el tercio apical (Fig. 15). Borde anterior de la frente con dos tubérculos mediofrontales secundarios, rudimentarios o grandes (Fig. 18)	**5**
4	Diente dorsal mandibular con una expansión cóncava (en vista dorsal). Mesosternón liso, sin puntos sobre la cicatriz mesosternal. Longitud total 16.0–19.5 mm	***Paxillus leachi* MacLeay**
–	Diente dorsal mandibular delgado, sin expansión cóncava. Mesosternón con puntos sobre la cicatriz mesosternal y más allá. Longitud total 14.0–16.0 mm	***Paxillus camerani* (Rosmini)**
5	Cicatriz mesosternal oval, poco marcada y brillante. Foseta metasternal y epipleura glabras. Longitud total 18.0–19.0 mm	***Paxillus forsteri* Luederwaldt**
–	Cicatriz mesosternal alargada, bien definida y opaca. Foseta metasternal y epipleura pubescentes	**6**
6	Primer artejo de la maza antenal reducido. Longitud total 22.7–23.1 mm (Fig. 18)	***Paxillus martinezi* Jiménez-Ferbans and Reyes-Castillo**
–	Primer artejo de la maza antenal no reducido, de largo similar al segundo. Longitud total 18.5–19.5 mm	***Paxillus pleuralis* Luederwaldt**
7	Borde frontal anterior recto o casi recto, sin tubérculos mediofrontales secundarios. Tubérculo central corto, con ápice no libre (fusionado a la frente y quillas frontales) (Fig. 19) (subgénero *Pertinax*)	**8**
–	Borde frontal anterior con uno o dos tubérculos mediofrontales secundarios (Figs 20–21); si no, entonces el tubérculo central con ápice muy libre (alcanzando o casi alcanzando el borde frontal anterior)	**15**
8	Maza antenal con cinco lamelas, las dos primeras reducidas. Longitud total 22.0–25.0 mm	**Passalus (Pertinax) rhodocanthopoides**
–	Maza antenal con tres lamelas (Fig. 19)	**9**
9	Alas reducidas (braquíptero) (Fig. 7)	**10**
–	Alas desarrolladas (macróptero)	**13**
10	Tubérculos lateroposteriores de mayor tamaño que el tubérculo central (Fig. 1). Humeri densamente pubescentes. Longitud total 32.8–36.8mm	**Passalus (Pertinax) bolivianus sp. nov.**
–	Tubérculos lateroposteriores más pequeños que el tubérculo central (Figs 5–7). Humeri glabros	**11**
11	Tubérculos internos unidos a tubérculos mediofrontales por una quilla débil. Área frontal, entre quillas frontales, no dividida longitudinalmente por un surco (Figs 6, 9). Parte media basal del mentón glabra (Fig. 8). Talla grande, longitud total 45.0–46.0 mm	**Passalus (Pertinax) canoi sp. nov.**
–	Tubérculos internos no unidos a tubérculos mediofrontales por una quilla (Fig. 5). Área frontal dividida longitudinalmente por un surco, desde el borde anterior hasta la base del mamelón cefálico. Parte media basal del mentón pubescente. Talla mediana (31.0–32.0 mm)	**12**
12	Borde frontal anterior con fuerte hendidura media, insinuando dientes mediofrontales secundarios (Fig. 5). Longitud total 31.3 mm	**Passalus (Pertinax) gonzalezae sp. nov.**
–	Borde frontal anterior sin hendidura media. Longitud total 32.0 mm	**Passalus (Pertinax) nodifrons Dibb**
13	Ápice del tubérculo central ligeramente libre, con solo una pequeña porción despegada de la frente. Longitud total 31.0–33.0 mm	**Passalus (Pertinax) catharinae Gravely**
–	Ápice del tubérculo central no libre, unido a la frente (Fig. 19)	**14**
14	Humeri con pubescencia escasa en la base. Talla pequeña (25.1–28.0 mm)	**Passalus (Pertinax) morio Percheron**
–	Humeri glabros. Talla grande (42.2–44.3 mm) (Fig. 19)	**Passalus (Pertinax) convexus Dalman**
15	Borde frontal anterior con un tubérculo mediofrontal secundario. Proceso hipostomal con un surco mate sobre el ápice	**Passalus (Mitrorhinus) lunaris (Kaup)**
–	Borde frontal anterior con dos tubérculos mediofrontales secundarios; si el el borde es recto, sin tubérculos, entonces el tubérculo central es muy libre (grupo "Petrejus"). Proceso hipostomal sin surco sobre el ápice (subgénero *Passalus*)	**16**
16	Borde frontal anterior con dos tubérculos mediofrontales secundarios, contiguos en su base	**17**
–	Borde frontal anterior sin tubérculos mediofrontales secundarios o con dos tubérculos separados en sus bases	**18**
17	Tubérculos mediofrontales secundarios grandes, fusionados entre si en casi toda su extensión. Áreas lateroposfrontales glabras. Longitud total 24.3–27.0 mm	**Passalus (Passalus) barrus Boucher and Reyes-Castillo**
–	Tubérculos mediofrontales secundarios pequeños, solo contiguos en su base. Áreas lateroposfrontales pubescentes. Longitud total 31.1–33.0 mm	**Passalus (Passalus) abortivus Percheron**
18	Tubérculo central con ápice muy libre, alcanzando o sobrepasando el borde frontal anterior. Tubérculos mediofrontales ausentes o rudimentarios (grupo “Petrejus”)	**19**
–	Tubérculo central con ápice no libre o ligeramente libre (Fig. 21); si es muy libre (alcanzando el borde anterior), entonces el metasternón está densamente pubescente (parte anterolateral). Tubérculos mediofrontales siempre presentes (Figs 20–21) (grupo “Neleus”)	**22**
19	Tubérculo central sobrepasando ampliamente el margen de la frente, fusionado a la parte media de la cabeza, casi hasta el borde anterior. Longitud total 24.0 mm	**Passalus (Passalus) pugionifer Kuwert**
–	Tubérculo central no fusionado a la parte media de la cabeza	**20**
20	Tubérculo central con concavidad en el ápice. Talla grande (40.0–51.0 mm)	**Passalus (Passalus) armatus Perty**
–	Tubérculo central sin concavidad en el ápice. Talla mediana (23.0–30.0 mm)	**21**
21	Tubérculo central con surco marcado en la base. Humeri pubescentes. Longitud total 30.0 mm	**Passalus (Passalus) inca Zang**
–	Tubérculo central sin surco en la base. Humeri glabros. Longitud total 23.0–30.0 mm	**Passalus (Passalus) pugionatus Burmeister**
22	Habitus opaco. Longitud total 39.5 mm	**Passalus (Passalus) opacus Gravely**
–	Habitus brillante	**23**
23	Foseta mesosternal glabra o con solo 1–3 sedas (*P. interruptus*)	**24**
–	Foseta mesosternal densamente pubescente	**27**
24	Maza antenal con cuatro lamelas, la cuarta muy reducida y tomentosa…..27.1–34.0 mm	**Passalus (Passalus) interstitialis Eschschltz**
–	Maza antenal con tres lamelas	**25**
25	Tubérculo central muy libre, alcanzando el borde anterior de la cabeza. Foseta lateral del pronoto pubescente. Longitud total 36.0–45.1 mm	**Passalus (Passalus) unicornis Lepeletier & Serville**
–	Tubérculo central solo ligeramente libre, nunca alcanzando el borde anterior de la cabeza. Foseta lateral del pronoto glabra	**26**
26	Surco marginal sobre último esternito abdominal incompleto. Talla grande (44.4–52.8 mm) (Fig. 20)	**Passalus (Passalus) interruptus (Linneo)**
–	Surco marginal sobre último esternito abdominal completo. Talla mediana a grande (29.1–42.0 mm) (Fig. 21)	**Passalus (Passalus) punctiger Lepeletier & Serville**
27	Ápice del tubérculo central muy libre, alcanzando el borde frontal anterior. Longitud total 33.0–38.0 mm	**Passalus (Passalus) coarctatus Percheron**
–	Ápice del tubérculo central no libre o apenas ligeramente libre, no alcanzando el borde frontal anterior. Longitud total 34.2–39.1 mm	**Passalus (Passalus) coniferus Eschscholtz**
28	Sutura frontoclipeal presente	**29**
–	Sutura frontoclipeal ausente (Fig. 10)	**30**
29	Maza antenal con tres lamelas. Longitud total 18.2–23.1 mm	***Popilius marginatus* (Percheron)**
–	Maza antenal con cuatro o cinco lamelas. Longitud total 17.0–21.0 mm	***Popilius tetraphyllus* (Eschscholtz)**
30	Borde anterior del labro profundamente cóncavo, con una excavación por detrás de la concavidad ("dorsal depression" *sensu*Marshall 2000). Longitud total 38.5–40.2 mm	***Verres furcilabris* (Eschschltz)**
–	Borde anterior del labro recto o ligeramente cóncavo o convexo, sin excavación por detrás del borde (*Veturius*)	**31**
31	Braquíptero. Longitud total 34.0–45.0 mm	**Veturius (Publius) spinipes (Zang)**
–	Macróptero (subgénero *Veturius*)	**32**
32	Mesosternón glabro (no incluyendo el ángulo anterior, que puede tener sedas cortas y dispersas)	**33**
–	Mesosternón con pubescencia abundante	**36**
33	Tubérculo central con ápice libre. Longitud total 36.0–40.0 mm	**Veturius (Veturius) libericornis Kuwert**
–	Tubérculo central con ápice no libre	**34**
34	Áreas lateroposfrontales pubescentes (raramente glabras). Metasternón con pubescencia más allá del borde anterior (cavidad metacoxal) y foseta lateral, alcanzando el área lateromedial del metasternón. Longitud total 37.0–49.0 mm	**Veturius (Veturius) standfussi Kuwert**
–	Áreas lateroposfrontales glabras. Metasternón con pubescencia restringida al borde anterior (cavidad metacoxal) y foseta lateral	**35**
35	Surco posfrontal (occipital *sensu*Reyes-Castillo 1970) ausente. Espolones superiores de meso y metatibias curvados. Longitud total 39.0–43.0 mm	**Veturius (Veturius) guntheri Kuwert**
–	Surco posfrontal presente. Espolones superiores de meso y metatibias rectos o casi rectos. Longitud total 39–46 mm	**Veturius (Veturius) yahua Boucher**
36	Áreas lateroposfrontales glabras. Longitud total 33.0–41.0 mm	**Veturius (Veturius) sinuosus (Drapiez)**
–	Áreas lateroposfrontales pubescentes	**37**
37	Áreas lateroposfrontales con sedas largas (2–15 sedas). Tubérculo central alto, en vista lateral mucho más elevado que tubérculos internos. Longitud total 30.0–37.0 mm	**Veturius (Veturius) boliviae Gravely**
–	Áreas lateroposfrontales con sedas cortas (2–10 sedas). Tubérculo central bajo, en vista lateral casi al mismo nivel que tubérculos internos. Longitud total 28.0–30.0 mm	**Veturius (Veturius) dreuxi Boucher**

**Figures 19–21.**
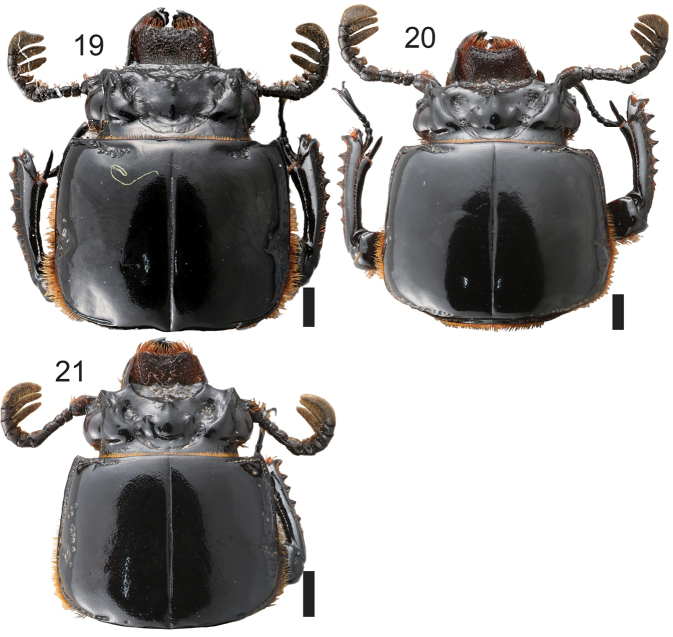
Head and pronotum: **19***Passalus convexus***20***Passalus interruptus***21***Passalus punctiger*. Scale bars: 2 mm.

## Discussion

Bolivia has a total area of 1,098,581 km^2^ and its territory includes a high variety of ecosystems. The country is divided in 12 ecoregions (Ibisch et al. 2003), of which, the Southwest Amazonia, Cerrado, Chiquitania, and Yungas seem to be suitable for Passalidae and we expected them to have high diversity of passalids. However, given its relative size, suitable climatic, ecological features, and mountainous areas, the real number of taxa occurring in the country is probably higher than the number of taxa registered to date.

The number of species known from Bolivia is small in comparison with other tropical countries of the New World. For example, Mexico, Guatemala, Colombia, and Brazil have more than 80 species recorded for each country (Fonseca and Reyes-Castillo 2004; Schuster 2006; Jiménez-Ferbans et al. 2018). Similarly, the number of endemic species is low, with *Veturius boliviae*, *Paxillus martinezi*, Passalus (Pertinax) nodifrons and Passalus (Passalus) opacus being the only endemic species of Bolivia.

Without doubt, the number of species of Bolivia is underestimated due to the lack of a systematic exploration of this country. Thus, more surveys are needed, especially in ecosystems such as montane forest and tropical rain forest, which normally harbor many species. Some departments with a domain of tropical rain forest have not been sampled for Passalidae; for example, Pando department has no records of passalid beetles, and for Beni department there are records of only 5 species. The majority of the specimens examined by us came from La Paz, Cochabamba and Santa Cruz departments, especially from mid-montane range locations, corresponding with the Yungas ecoregion. Several studies have reported this pattern in Passalidae, with a high level of richness at mid-mountain ranges (MacVean and Schuster 1981, Jiménez-Ferbans et al. 2010, Chamé-Vázquez et al. 2018). However, due to the extension of these departments, the amount of known species is still considered low, pointing out the need of sampling in the mid-range montane ecosystems of Bolivia.

### Reliability of the species records

From the total of 38 species listed above, we have studied material for 23 species. For the other 15 species, some authors have recorded specimens of all of them. However, three species can be discussed. The record of *P. unicornis* is based on a specimen recorded by Luederwaldt (1931b). However, Luedewaldt himself pointed out some differences of the Bolivian specimens regarding other specimens of *P. unicornis*. Likewise, the length of the specimen is too small and perhaps it corresponds to *P. coarctatus*, since these two species are commonly confused with each other.

*Passalus morio* has been recorded for Colombia, Guiana and Suriname; nonetheless, as far as we know, it is distributed mostly in the Atlantic Forest (Fonseca and Reyes-Castillo 2004; Jiménez-Ferbans et al. 2013), and its record for Bolivia must be confirmed.

Finally, the record of *Passalus catharinae* from Bolivia must be confirmed because no records of this species are available except for the original description. Its record for Bolivia is based on the interpretation of “Chaco” (Gravely 1918) as “Bolivia: Chaco” made by Hincks and Dibb (1935). A similar situation occurred with *Veturius sinuatosulcatus* Gravely. Hincks and Dibb (1935) recorded *V. sinuatosulcatus* from “Bolivia: Chaco”. However, Boucher (2006) stating that Hincks and Dibb (1935) must have misinterpreted the type locality “Chaco” as “Chaco, Bolivia”, since *V. sinuatosulcatus* (now synonym *V. sinuatocollis* sensu Boucher (2006)) does not occur in Bolivia. Then, probably the reference of “Chaco” by Gravely may not correspond to the Chao from Bolivia.

## Supplementary Material

XML Treatment for Passalus (Pertinax) bolivianus

XML Treatment for Passalus (Pertinax) gonzalezae

XML Treatment for Passalus (Pertinax) canoi
